# Illicit Drug Use and Risk for USA300 Methicillin-Resistant *Staphylococcus aureus* Infections with Bacteremia

**DOI:** 10.3201/eid1609.091802

**Published:** 2010-09

**Authors:** Kristen M. Kreisel, J. Kristie Johnson, O. Colin Stine, Michelle D. Shardell, Eli N. Perencevich, Alan J. Lesse, Fred M. Gordin, Michael W. Climo, Mary-Claire Roghmann

**Affiliations:** Author affiliations: University of Maryland, Baltimore, Maryland, USA (K.M. Kreisel, J.K. Johnson, O.C. Stine, M.D. Shardell, E.N. Perencevich, M.-C. Roghmann);; VA Maryland Health Care System, Baltimore (K.M. Kreisel, E.N. Perencevich, M.-C. Roghmann);; VA Western New York Healthcare System, Buffalo, New York, USA (A.J. Lesse);; University at Buffalo, Buffalo (A.J. Lesse);; Washington DC VA Medical Center, Washington, DC, USA (F.M. Gordin);; George Washington University, Washington (F.M. Gordin);; Hunter Holmes McGuire VA Medical Center, Richmond, VA, USA (M.W. Climo);; Medical College of Virginia at Virginia Commonwealth University, Richmond (M.W. Climo)

**Keywords:** Staphylococcus aureus, antimicrobial resistance, community-acquired infections, bacteremia, bacterial typing techniques, electrophoresis, bacteria, illicit drug use, research

## MEDSCAPE CME

Medscape, LLC is pleased to provide online continuing medical education (CME) for this journal article, allowing clinicians the opportunity to earn CME credit. This activity has been planned and implemented in accordance with the Essential Areas and policies of the Accreditation Council for Continuing Medical Education through the joint sponsorship of Medscape, LLC and Emerging Infectious Diseases. Medscape, LLC is accredited by the ACCME to provide continuing medical education for physicians. Medscape, LLC designates this educational activity for a maximum of 0.5 *AMA PRA Category 1 Credits*™. Physicians should only claim credit commensurate with the extent of their participation in the activity. All other clinicians completing this activity will be issued a certificate of participation. To participate in this journal CME activity: (1) review the learning objectives and author disclosures; (2) study the education content; (3) take the post-test and/or complete the evaluation at http://cme.medscape.com/viewpublication/30063; (4) view/print certificate.

## Learning Objectives

Upon completion of this activity, participants will be able to:

- Examine the association of illicit drug use with USA300 MRSA infection, including risk factors for acquisition and transmission.
- Describe characteristics of illicit drug users who acquire MRSA and the changing pattern of risk from 2004 to 2008 in the United States with implications for management and prevention.

## MEDSCAPE CME Editor

**Carol Snarey, MA, **Copyeditor, *Emerging Infectious Diseases. Disclosure: Carol Snarey has disclosed no relevant financial relationships.*

## MEDSCAPE CME AUTHOR

**Desiree Lie, MD, MSED, **Clinical Professor of Family Medicine, Director of Research and Faculty Development, University of California, Irvine at Orange, California. *Disclosure: Désirée Lie, MD, MSEd, has disclosed the following relevant financial relationship: served as a nonproduct speaker for "Topics in Health" for Merck Speaker Services.*

## AUTHORS

*Disclosures:* ***Kristen M. Kreisel, PhD; O. Colin Stine, PhD; Michelle D. Shardell, PhD; Alan J. Lesse, MD; Fred M. Gordin, MD;*** *and* ***Mary-Claire Roghmann, MD, MS,*** *have disclosed no relevant financial relationships.* ***J. Kristie Johnson, PhD,*** *has disclosed the following relationships: received grants for clinical research from Becton, Dickinson and Company.* ***Eli N. Perencevich, MD, MS,*** *has disclosed the following relationships: received grants for clinical support from Pfizer Inc.* ***Michael W. Climo, MD,*** *has disclosed the following relationships: served as an advisor or consultant for Biosynexus Incorporated; received grants for clinical research from Biosynexus Incorporated.*
