# Supplementary material for: Illicit Drug Use and Risk for USA300 Methicillin-Resistant Staphylococcus aureus Infections with Bacteremia
Source: Emerg Infect Dis. 2010 Sep;16(9):1419–27. doi: 10.3201/eid1609.091802 (PMC3294968; doi:10.3201/eid1609.091802)
Supplement: Related texts

# Research article: Illicit Drug Use and Risk for USA300 Methicillin-Resistant *Staphylococcus aureus* Infections with Bacteremia

**DOI:** 10.3201/eid1609.091802

**Authors:** Kristen M. Kreisel, J. Kristie Johnson, O. Colin Stine, Michelle D. Shardell, Eli N. Perencevich, Alan J. Lesse, Fred M. Gordin, Michael W. Climo, Mary-Claire Roghmann

**Affiliations:** Author affiliations: University of Maryland, Baltimore, Maryland, USA (K.M. Kreisel, J.K. Johnson, O.C. Stine, M.D. Shardell, E.N. Perencevich, M.-C. Roghmann);; VA Maryland Health Care System, Baltimore (K.M. Kreisel, E.N. Perencevich, M.-C. Roghmann);; VA Western New York Healthcare System, Buffalo, New York, USA (A.J. Lesse);; University at Buffalo, Buffalo (A.J. Lesse);; Washington DC VA Medical Center, Washington, DC, USA (F.M. Gordin);; George Washington University, Washington (F.M. Gordin);; Hunter Holmes McGuire VA Medical Center, Richmond, VA, USA (M.W. Climo);; Medical College of Virginia at Virginia Commonwealth University, Richmond (M.W. Climo)

## Abstract

To assess the association of illicit drug use and USA300 methicillin-resistant *Staphylococcus aureus* (MRSA) bacteremia, a multicenter study was conducted at 4 Veterans Affairs medical centers during 2004–2008. The study showed that users of illicit drugs were more likely to have USA300 MRSA bacteremia (in contrast to bacteremia caused by other *S. aureus* strains) than were patients who did not use illicit drugs (adjusted relative risk 3.0; 95% confidence interval 1.9–4.4). The association of illicit drug use with USA300 MRSA bacteremia decreased over time (p = 0.23 for trend). Notably, the proportion of patients with USA300 MRSA bacteremia who did not use illicit drugs increased over time. This finding suggests that this strain has spread from users of illicit drugs to other populations.

Infections caused by community-associated methicillin-resistant *Staphylococcus aureus* (CA-MRSA) are increasing. Outbreaks have been described in a variety of populations, including sports teams, men who have sex with men, prisoners, and children (*1**–**10*). The USA300 MRSA clone has been recognized as the most common strain causing CA-MRSA infections (*11*).

CA-MRSA was first reported in illicit drug users in Detroit in 1980 (*12*). The drug-using population has been identified as a reservoir of CA-MRSA (*13*). Because of the repeated injection or inhalation of drugs, the opportunity for a person to cause and spread infection with one’s own colonizing strain is multiplied (*13**,**14*). Skin and soft tissue infections are the most common infections in illicit drug users; the USA300 MRSA strain is the cause of up to 75% of these infections (*13**–**15*). Once this strain colonizes or otherwise infects a person, it can then enter the patient’s bloodstream and become a potentially life-threatening bloodstream infection.

If admitted to the hospital, illicit drug–using patients with a USA300 MRSA infection complicated by bacteremia serve as a potential reservoir for transmission to other patient populations. This mechanism may be contributing to the replacement of other MRSA strains typically associated with nosocomial infections by USA300 MRSA and may aid this strain in becoming the predominant isolate causing MRSA infections in both healthcare and community settings (*16**,**17*). The objective of this study was to evaluate the association of illicit drug use with USA300 MRSA bacteremia and whether the association is static or has changed over a 5-year period as the USA300 MRSA epidemic has progressed.

## Methods

### Study Design and Population

A multicenter retrospective cohort study was conducted by using patients from the population of veterans enrolled from January 2004 through June 2008 at the Veterans Affairs medical centers (VAMCs) in Baltimore, Maryland; Washington, DC; Buffalo, New York; and Richmond, Virginia. Patients who used illicit drugs were compared with those who did not use illicit drugs with respect to having USA300 MRSA bacteremia vs. bacteremia caused by all other types of *S. aureus* (this group includes non-USA300 MRSA and methicillin-susceptible *S. aureus* [MSSA]). Inclusion criteria for patients included in the study were the following: 1) age >18 years, 2) enrollment in patient care services at 1 of the 4 VAMCs, 3) a positive blood culture for *S. aureus*, 4) having first known invasive infection caused by *S. aureus,* and 5) having a bacterial isolate from the infection available for testing. We excluded patients from the analysis for whom the infection was found to be polymicrobial, or for whom the bacteremic episode was considered to be clinically insignificant (i.e., the patient did not have clinical symptoms consistent with the presence of infection, such as fever) (*18*). The Institutional Review Boards at all participating sites approved this protocol.

### Data Collection and Definitions

Data were collected from patient electronic medical records, which included administrative coding data. An infection control nurse, who was blinded to the outcome of each patient, conducted chart review by using a standardized form. Illicit drug use was defined by International Classification of Diseases, 9th revision (ICD-9), codes indicating abuse of or dependence on cocaine (ICD-9 codes: 304.21, 304.20, 304.23, 305.61, 305.62, 305.60) or opioids (ICD-9 codes: 304.01, 304.71, 304.00, 305.51, 304.73), designated at any hospitalization up to 1 year prior to the time of the patient’s presentation. The electronic medical record of any patient identified as an illicit drug user was further evaluated to determine whether the abuse was by injection.

An infection was defined as nosocomial if the patient’s blood culture was positive for *S. aureus* >48 hours after hospital admission, if the patient was transferred from another healthcare facility, or if the infection was central-line associated. Infections were defined as central-line associated if a primary source was identified (i.e., no other source of infection could be found) and if the patient had a central line in place for 48 hours before the onset of bacteremia (*19*). If the patient had pneumonia, a skin and soft tissue infection, a urinary tract infection, or some other source that could explain the basis for infection, the bacteremia was defined as a secondary infection. Infective endocarditis was defined by using the modified Duke criteria (*20*).

Information on risk factors for MRSA acquisition and infection was also obtained. They included whether the patient had been hospitalized, had surgery, resided in a long-term care facility, or had undergone hemodialysis in the year before infection, as well as if any foreign medical device was present at the time of infection or if the patient had been previously colonized or infected with MRSA. The presence of HIV was assessed, as well as the presence of comorbid conditions to calculate each patient’s Charlson score (*21*).

### Laboratory Evaluations

All *S. aureus* isolates were sent for testing to the Baltimore VAMC. *S. aureus* was confirmed by standard microbiologic techniques. Any isolate with growth on oxacillin screen agar was defined as MRSA; any isolate without growth on this agar was defined as MSSA. All *S. aureus* isolates were screened for the presence of the Panton-Valentine leukocidin gene (PVL; *luk-F-PV, luk-S-PV*), as previously described (*22*). Further screening for the presence of the arginine catabolic mobile element gene (ACME; *arc*A) and sequencing of the protein A (*spa*) gene hypervariable region was also performed on MRSA isolates only, as previously described (*23**,**24*). Patient sequences were compared with sequences found in the Ridom *spa* Server (www.ridom.de/spaserver).

USA300 MRSA isolates were identified by using an algorithm previously described (*5*). Any MRSA isolate that tested positive for the genes for PVL and ACME, and was *spa* type motif MBQBLO, was classified as USA300 MRSA. These isolates were confirmed as USA300 MRSA by pulsed-field gel electrophoresis (PFGE) by using a 24% random sample (13/55 suspected USA300 MRSA isolates were tested) (*11*). MRSA isolates that were negative for all 3 genetic factors were classified as non-USA300 MRSA. A 26% random sample of the non-USA300 MRSA isolates was also confirmed by PFGE (27/103 suspected non-USA300 MRSA isolates were tested) (*11*). All MRSA isolates testing positive for at least 1 of the 3 (PVL, ACME, or *spa* type motif MBQBLO) were further characterized by PFGE to determine whether any were the USA300 MRSA strain. If pulsed-field type USA300 by PFGE, the isolates were classified as USA300 MRSA. All other MRSA isolates (non-USA300 MRSA) and MSSA isolates were classified as “all other *S. aureus*.” The Fingerprinting II software was used to analyze the electronic images of the gels (Bio-Rad Laboratories, Hercules, CA, USA). The banding patterns of each isolate were compared with the USA PFGE types described by McDougal et al. (*11*); the similarity between isolates was assessed by using the criteria established by Tenover et al. (*25*)

### Statistical Analysis

Data were analyzed by using the SAS statistical software package, version 9.1 (SAS Institute Inc., Cary, NC, USA). The Pearson χ^2^ or Fisher exact tests were used to compare categorical variables, and the Student *t* test or Wilcoxon signed-rank test was used to compare continuous variables. A p value <0.05 was considered significant. Unadjusted relative risks (RR) were calculated to estimate the association between illicit drug use and USA300 MRSA bacteremia. Stratified analyses were conducted to test for effect modification and confounding; any variable with a Breslow-Day p value <0.05 was considered significant, while a 10% difference between the unadjusted and adjusted RR was used to identify confounding. A binomial regression using a log link was fit to estimate the association between illicit drug use and USA300 MRSA bacteremia, adjusting for identified confounders and/or effect modifiers.

## Results

We identified 300 patients with *S. aureus* bacteremia at the 4 participating sites during the study period. Strains having all 3 genetic factors (PVL, ACME, and *spa* type motif MBQBLO) were classified as USA300 MRSA, and a random sample of these isolates showed 100% sensitivity and specificity by PFGE. Isolates with none of these genetic factors were classified as non-USA300 MRSA, and a random sample of these isolates also showed 100% sensitivity and specificity by PFGE. Isolates with 1 or 2 of the genetic factors were also designated as non-USA300 MRSA; 18% of these isolates were found to be USA300 MRSA by PFGE, resulting in 100% sensitivity and 82% specificity of the laboratory algorithm for identifying USA300 MRSA. Sixty-seven (22%) of the infections were caused by USA300 MRSA, 117 (39%) by non-USA300 MRSA, and 116 (39%) by MSSA.

Patient and infection characteristics of the study population are presented in Table 1 and Table 2. Of all patients with *S. aureus* bacteremia, 22 (7%) were illicit drug users, 13 (59%) with injection drugs. The patients had a mean age of 68 years and were almost all male (98%). Infections were classified as nosocomial in 172 (57%) patients, 83 (48%) of which were central-line associated. Sixteen (5%) patients were infected with HIV and 80 (27%) had a previous episode of colonization or infection with MRSA.

**Table 1:** Isolate and patient characteristics for 300 veterans who had *Staphylococcus aureus* bacteremia at 4 Veterans Affairs medical centers, USA, 2004–2008*

Characteristic	No. (%)
*S. aureus* isolate characteristics	
USA300 MRSA	67 (22)
All other *S. aureus*	233 (78)
Non-USA300 MRSA	117 (39)
MSSA	116 (39)
Patient characteristics	
Age, y, mean ± SD	68 ± 13
Sex	
M	295 (98)
F	5 (2)
Race	
Black	95 (32)
Other	205 (68)
Year of presentation†	
Late	169 (56)
Early	131 (44)
Charlson score, mean ± SD	4.7 ± 3.0
HIV infection	
Yes	16 (5)
No	284 (95)
Illicit drug use	
Yes	22 (7)
No	278 (93)
History of colonization or infection with MRSA	
Yes	80 (27)
No	220 (73)
Hospitalized in year before infection	
Yes	195 (65)
No	105 (35)
Surgery in year before infection	
Yes	109 (36)
No	191 (64)
Residence in LTCF in year before infection	
Yes	34 (11)
No	266 (89)
Renal failure in year before infection	
Yes	114 (38)
No	186 (62)

*Values are no. (%) except as indicated. MRSA, methicillin-resistant *S. aureus*; MSSA, methicillin-susceptible *S. aureus*; LTCF, long-term care facility. †Year of presentation stratified into 2 periods: early (January 1, 2004–March 31, 2006) and late (April 1, 2006–June 30, 2008).

**Table 2:** Infection characteristics for 300 veterans who had *Staphylococcus aureus* bacteremia at 4 Veterans Affairs medical centers, USA, 2004–2008

Infection characteristic	Value
Nosocomial infection	
Yes	172 (57)
No	128 (43)
Central line at time of infection	
Yes	83 (28)
No	217 (72)
Permanent hardware at time of infection	
Yes	88 (29)
No	212 (71)
Source of infection	
Primary	126 (42)
Secondary	174 (58)
Infection complicated by endocarditis*	
Yes	38 (13)
No	262 (87)
Infection complicated by pneumonia†	
Yes	45 (15)
No	255 (85)

*Data missing for 4 patients (1%). †Data missing for 11 patients (4%).

Compared with patients who did not use illicit drugs, illicit drug users were younger (mean age 51 vs. 69; p<0.0001; Table 3) and more likely to be African American (17% vs. 3%; p<0.0001). Illicit drug users were more likely to have HIV (38% vs. 6%; p<0.0001) or endocarditis (16% vs. 6%; p = 0.04) and less likely to have acquired their infection nosocomially (3% vs. 13%; p = 0.003) than patients who did not use illicit drugs. Illicit drug users were significantly more likely to have a bacteremic infection caused by USA300 MRSA than by all other *S. aureus* strains, compared with patients who did not use illicit drugs (RR 3.04, 95% confidence interval [CI] 1.99–4.64; p<0.0001; Table 4). Age (mean age 63 vs. 69 years; p = 0.0004) and a nosocomial acquisition of infection (RR 0.44, 95% CI 0.29–0.69; p = 0.0002) were both negatively associated with USA300 MRSA bacteremia.

**Table 3:** Patient and infection characteristics for 300 veterans with *Staphylococcus aureus* bacteremia and association with illicit drug use at 4 Veterans Affairs medical centers, USA, 2004–2008*

Variable	Used illicit drugs,† n = 22	No illicit drug use,† n = 278	p value‡
Patient characteristics			
Age, y, mean ± SD	51 ± 5	69 ± 12	<0.0001
Sex			
M	20 (7)	275 (93)	0.05
F	2 (40)	3 (60)	
Race			
Black	16 (17)	79 (83)	<0.0001
Other	6 (3)	199 (97)	
Year of presentation§			
Late	10 (6)	159 (94)	0.29
Early	12 (9)	119 (91)	
Charlson score, mean ± SD	5.4 ± 4.1	4.7 ± 2.9	0.28
HIV infection			
Yes	6 (38)	10 (62)	<0.0001
No	16 (6)	268 (94)	
History of colonization or infection with MRSA			
Yes	5 (6)	75 (94)	0.66
No	17 (8)	203 (92)	
Hospitalized in year before infection			
Yes	15 (8)	180 (92)	0.75
No	7 (7)	98 (93)	
Surgery in year before infection			
Yes	1 (1)	108 (99)	<0.0001
No	21 (11)	170 (89)	
Residence in LTCF in year before infection			
Yes	3 (9)	31 (91)	0.73
No	19 (7)	247 (93)	
Renal failure in year before infection			
Yes	14 (8)	172 (92)	0.87
No	8 (7)	106 (93)	
Infection characteristics			
Nosocomial infection			
Yes	6 (3)	166 (97)	0.003
No	16 (13)	112 (87)	
Central line at time of infection			
Yes	5 (6)	78 (94)	0.59
No	17 (8)	200 (92)	
Permanent hardware at time of infection			
Yes	4 (5)	84 (95)	0.33
No	18 (8)	194 (92)	
Source of infection			
Primary	10 (8)	116 (92)	0.73
Secondary	12 (7)	162 (93)	
Infection complicated by endocarditis			
Yes	6 (16)	32 (84)	0.04
No	16 (6)	242 (94)	
Infection complicated by pneumonia			
Yes	3 (7)	42 (93)	1.00
No	19 (7)	236 (93)	

*MRSA, methicillin-resistant *S. aureus*; LTCF, long-term care facility. †Values are no. (%) except as indicated. ‡Calculated by using Pearson χ^2^ or Fisher exact tests for categorical variables, Student *t* test or Wilcoxon signed-rank test for continuous variables. §Year of presentation stratified into 2 periods: early (January 1, 2004–March 31, 2006) and late (April 1, 2006–June 30, 2008)**.**

**Table 4:** Patient and infection characteristics for 300 veterans with *Staphylococcus aureus* bacteremia and association with USA300 MRSA at 4 Veterans Affairs medical centers, USA, 2004–2008*

Variable	USA300 MRSA bacteremia,† n = 67	Bacteremia due to all other *S. aureus,*† n = 233	p value‡
Patient characteristics			
Age, y, mean ± SD	63 ± 12	69 ± 13	0.0004
Sex			
M	65 (22)	230 (78)	0.31
F	2 (40)	3 (60)	
Race			
Black	34 (36)	61 (64)	0.0001
Other	33 (16)	172 (84)	
Year of presentation§			
Late	45 (27)	124 (73)	0.04
Early	22 (17)	109 (83)	
Charlson score, mean ± SD	5.2 ± 3.6	4.6 ± 2.8	0.19
HIV infection			
Yes	5 (31)	11 (69)	0.36
No	62 (22)	222 (78)	
Illicit drug use			
Yes	13 (59)	9 (41)	<0.0001
No	54 (19)	224 (81)	
History of colonization or infection with MRSA*			
Yes	21 (26)	59 (74)	0.33
No	46 (21)	174 (79)	
Hospitalized in year before infection			
Yes	40 (21)	155 (79)	0.30
No	27 (26)	78 (74)	
Surgery in year before infection			
Yes	17 (16)	92 (84)	0.03
No	50 (26)	141 (74)	
Residence in LTCF in year before infection			
Yes	10 (29)	24 (71)	0.29
No	57 (21)	209 (79)	
Renal failure in year before infection			
Yes	42 (23)	144 (77)	0.90
No	25 (22)	89 (78)	
Infection characteristics			
Nosocomial infection			
Yes	25 (15)	147 (85)	0.0002
No	42 (33)	86 (67)	
Central line at time of infection			
Yes	11 (13)	72 (87)	0.02
No	56 (26)	161 (74)	
Permanent hardware at time of infection			
Yes	20 (33)	68 (77)	0.92
No	48 (28)	126 (72)	
Source of infection			
Primary	19 (15)	107 (85)	0.01
Secondary	48 (28)	126 (72)	
Infection complicated by endocarditis			
Yes	9 (24)	29 (76)	0.83
No	57 (22)	201 (78)	
Infection complicated by pneumonia			
Yes	8 (18)	37 (82)	0.40
No	57 (23)	186 (77)	

*MRSA, methicillin-resistant *S. aureus*, LTCF, long-term care facility. †Values are no. (%) except as indicated. ‡Calculated by using Pearson χ^2^ or Fisher exact tests for categorical variables, Student *t* test or Wilcoxon signed-rank test for continuous variables. §Year of presentation stratified into 2 periods: early (January 1, 2004–March 31, 2006) and late (April 1, 2006–June 30, 2008)**.**

Using binomial regression, illicit drug users were significantly more likely to have USA300 MRSA bacteremia compared with patients not using illicit drugs, controlling for year of presentation (adjusted RR [aRR] 3.00, 95% CI 1.88–4.36; p<0.0001; Table 5). This result was due to an increase in the proportion of *S. aureus* bacteremic infections caused by the USA300 MRSA strain in patients who did not use illicit drugs over the study period, while the proportion in patients using illicit drugs remained relatively stable after 2004. Stratified by year of presentation (categorized as early [January 1, 2004–March 31, 2006] vs. late [April 1, 2006–June 30, 2008] years of presentation), the association of illicit drug use with USA300 MRSA bacteremia decreased over the study period (RR in early years of presentation 4.63, 95% CI 2.36–9.07; p<0.0001; RR for late years of presentation 2.45, 96% CI 1.38–4.35; p = 0.02). The association of illicit drug use and USA300 MRSA bacteremia weakened over the study period, although the trend was not statistically significant (p = 0.23 for trend over time; Figure).

**Table 5:** Independent risk factors for USA300 MRSA bacteremia among 300 veterans at 4 Veterans Affairs medical centers, USA, 2004–2008*

Variable	Adjusted RR (95% CI)	p value
Illicit drug use	3.00 (1.88–4.36)	<0.0001
Late year of presentation†	1.56 (1.03–2.48)	0.04

*MRSA, methicillin-resistant *Staphylococcus aureus*; RR, relative risk; CI, confidence interval. †Year of presentation stratified into 2 periods: early (January 1, 2004–March 31, 2006) and late (April 1, 2006–June 30, 2008)**.**

**Figure:** Association between illicit drug use and USA300 methicillin-resistant *Staphylococcus aureus* bacteremia among 300 veterans at 4 Veterans Affairs medical centers, USA, 2004–2008 (generalized linear model p value for trend over time = 0.23). †No illicit drug users had a bacteremic infection caused by USA300 MRSA in 2006.

## Discussion

In this multicenter study, illicit drug users were more likely to have a bacteremic infection caused by USA300 MRSA than any other type of *S. aureus*, regardless of when the patient’s infection occurred. The data were consistent with a decrease in the association over the study period, from a RR of 4.63 in the early years of the study to a RR of 2.45 in the late years of the study.

The finding that the association between illicit drug use and USA300 MRSA bacteremia declined over the study period is clinically important. This illustrates that, although USA300 MRSA infections emerged in drug users, this epidemic is now spreading to other populations. The illicit drug–using population has been recognized as a reservoir for USA300 MRSA (*14*). The spread to other populations observed in this study could be the result of transmission and dissemination of the strains among at-risk patients in the community, as suggested by the fact that 43% of cases of bacteremia were not classified as nosocomial infections in this population. This means that patients who have bacteremia could have acquired the bacteria during a previous healthcare exposure or in the community. In contrast, illicit drug users who are colonized or infected with USA300 MRSA could serve as a reservoir for transmission to other patient populations while hospitalized for their infections (*12**–**14**,**26**,**27*). Further investigation is warranted.

The increased risk of acquiring an infection caused by USA300 MRSA among illicit drug users found in this study is consistent with the findings of other studies. Gilbert et al. reported that the incidence of USA300 MRSA was higher among high-risk case-patients (defined as including a history of illicit drug use) than low-risk case-patients (*28*). In a study of the New York State Prison System, inmates with drug charges were more likely to have an infection. The USA300 MRSA strain was the predominant clone that caused infections in inmates (*29*). Although our study used different sampling methods and slightly different case definitions than previous studies, the conclusions are the same. Illicit drug users are at increased risk for an infection caused by USA300 MRSA compared with persons who do not use illicit drugs.

The association between illicit drug use and USA300 MRSA bacteremia could be explained by the fact that *S. aureus* infections are a common complication of drug use. Drug users are colonized with *S. aureus* more often than persons who do not use drugs (*13**,**14*). Colonization is a main risk factor for infection; the patient is usually infected with his own colonizing strain. In addition, invasive infections can occur from transfer of the colonizing strain directly into the patient’s bloodstream (*13**,**14*). If USA300 MRSA is the colonizing strain, the frequent inhalation or injection of drugs could transfer this strain into the bloodstream to cause a life-threatening invasive infection.

Our study has some limitations. First, data regarding illicit drug use at the various VAMCs is based on self-report. Information regarding drug use is of a sensitive nature and, therefore, subject to recall bias. Also, to locate in the patient’s electronic medical record whether the drugs were injected depended on whether the healthcare professional specifically asked and made note of this, a situation which is prone to information bias. Second, the ICD-9 codes used to define illicit drug use are imperfect measurements because they are used for insurance billing, rather than clinical purposes. Also, if any illicit drug–using patients were not hospitalized in the year before enrollment, or if drug-using patients did not report drug use during the hospitalization of interest, drug use would have been misclassified. In addition, using ICD-9 codes to define drug use up to 1 year before admission may not accurately measure current patterns of drug use in patients; the result would be a differential misclassification of exposure and could overestimate or underestimate the true association. Third, the study population was comprised only of veterans. The use of such a distinct population could reduce the generalizability of these findings. However, the relationship of illicit drug use with USA300 MRSA among veterans would likely not differ from the relationship among nonveterans, so the results should still be generalizable. Finally, USA300 MRSA isolates could have been misclassified with the use of our laboratory algorithm; however, the validation of isolates by PFGE, which showed a high sensitivity and specificity, makes this unlikely.

This study has several strengths. First, the use of more than 1 study site helped improve the generalizability of these findings. For example, we evaluated whether any of the associations observed may have been due to differences between the 4 VAMCs (this analysis was in response to a finding that patients from the Buffalo VAMC were less likely to be illicit drug users and less likely to have an infection due to USA300 MRSA). After excluding all patients from the Buffalo VAMC from the analysis, we found no difference from the results of the entire cohort (data not shown); therefore, we chose to present the combined data. Additionally, the electronic medical record system used throughout the Veterans Health Administration is known to be a valuable asset because of the completeness of data and for the amount of time it has stored information. The use of this system for chart review helped decrease selection and information bias. Finally, the findings of this study are strengthened because we could provide molecular typing data for each of the isolates.

In conclusion, the data from this study showed that illicit drug users are more likely to acquire a bacteremic infection caused by USA300 MRSA than by all other *S. aureus* strains. The decrease observed in the association of illicit drug use and USA300 MRSA bacteremia over the study period suggests that the USA300 MRSA epidemic is now spreading from illicit drug users to other patient populations. Focusing infection control efforts on high-risk groups such as illicit drug users might slow the progression of the USA300 MRSA epidemic in areas of the country where the association between illicit drug use and USA300 is still high.

## References

[1] Centers for Disease Control and Prevention. Four pediatric deaths from community-acquired methicillin-resistant *Staphylococcus aureus*—Minnesota and North Dakota, 1997–1999. JAMA. 1999;282:1123–5. 10.1001/jama.282.12.112310501104

[2] Outbreaks of community-associated methicillin-resistant *Staphylococcus aureus* skin infections—Los Angeles County, California, 2002–2003. MMWR Morb Mortal Wkly Rep. 2003;52:88.12588006

[3] Community-associated methicillin-resistant *Staphylococcus aureus* infections in Pacific Islanders—Hawaii, 2001–2003. MMWR Morb Mortal Wkly Rep. 2004;53:767–70.15329653

[4] Groom AV, Wolsey DH, Naimi TS, Smith K, Johnson S, Boxrud D, Community-acquired methicillin-resistant *Staphylococcus aureus* in a rural American Indian community. JAMA. 2001;286:1201–5. 10.1001/jama.286.10.120111559265

[5] Herold BC, Immergluck LC, Maranan MC, Lauderdale DS, Gaskin RE, Boyle-Vavra S, Community-acquired methicillin-resistant *Staphylococcus aureus* in children with no identified predisposing risk. JAMA. 1998;279:593–8. 10.1001/jama.279.8.5939486753

[6] Kazakova SV, Hageman JC, Matava M, Srinivasan A, Phelan L, Garfinkel B, A clone of methicillin-resistant *Staphylococcus aureus* among professional football players. N Engl J Med. 2005;352:468–75. 10.1056/NEJMoa04285915689585

[7] Lee NE, Taylor MM, Bancroft E, Ruane PJ, Morgan M, McCoy L, Risk factors for community-associated methicillin-resistant *Staphylococcus aureus* skin infections among HIV-positive men who have sex with men. Clin Infect Dis. 2005;40:1529–34. 10.1086/42982715844078PMC6784309

[8] Lindenmayer JM, Schoenfeld S, O’Grady R, Carney JK. Methicillin-resistant *Staphylococcus aureus* in a high school wrestling team and the surrounding community. Arch Intern Med. 1998;158:895–9. 10.1001/archinte.158.8.8959570176

[9] Naimi TS, LeDell KH, Como-Sabetti K, Borchardt SM, Boxrud DJ, Etienne J, Comparison of community- and health care–associated methicillin-resistant *Staphylococcus aureus* infection. JAMA. 2003;290:2976–84. 10.1001/jama.290.22.297614665659

[10] Stacey AR, Endersby KE, Chan PC, Marples RR. An outbreak of methicillin resistant *Staphylococcus aureus* infection in a rugby football team. Br J Sports Med. 1998;32:153–4. 10.1136/bjsm.32.2.1539631224PMC1756087

[11] McDougal LK, Steward CD, Killgore GE, Chaitram JM, McAllister SK, Tenover FC. Pulsed-field gel electrophoresis typing of oxacillin-resistant *Staphylococcus aureus* isolates from the United States: establishing a national database. J Clin Microbiol. 2003;41:5113–20. 10.1128/JCM.41.11.5113-5120.200314605147PMC262524

[12] Saravolatz LD, Markowitz N, Arking L, Pohlod D, Fisher E. Methicillin-resistant *Staphylococcus aureus*. Epidemiologic observations during a community-acquired outbreak. Ann Intern Med. 1982;96:11–6.705368310.7326/0003-4819-96-1-11

[13] Gordon RJ, Lowy FD. Bacterial infections in drug users. N Engl J Med. 2005;353:1945–54. 10.1056/NEJMra04282316267325

[14] Lowy FD, Miller M. New methods to investigate infectious disease transmission and pathogenesis—*Staphylococcus aureus* disease in drug users. Lancet Infect Dis. 2002;2:605–12. 10.1016/S1473-3099(02)00395-X12383610

[15] Moran GJ, Krishnadasan A, Gorwitz RJ, Fosheim GE, McDougal LK, Carey RB, Methicillin-resistant *S. aureus* infections among patients in the emergency department. N Engl J Med. 2006;355:666–74. 10.1056/NEJMoa05535616914702

[16] Diep BA, Sensabaugh GF, Somboona NS, Carleton HA, Perdreau-Remington F. Widespread skin and soft-tissue infections due to two methicillin-resistant *Staphylococcus aure*us strains harboring the genes for Panton-Valentine leucocidin. J Clin Microbiol. 2004;42:2080–4. 10.1128/JCM.42.5.2080-2084.200415131173PMC404676

[17] Seybold U, Kourbatova EV, Johnson JG, Halvosa SJ, Wang YF, King MD, Emergence of community-associated methicillin-resistant *Staphylococcus aureus* USA300 genotype as a major cause of health care–associated blood stream infections. Clin Infect Dis. 2006;42:647–56. 10.1086/49981516447110

[18] Soriano A, Martinez JA, Mensa J, Marco F, Almela M, Moreno-Martínez A, Pathogenic significance of methicillin resistance for patients with *Staphylococcus aureus* bacteremia. Clin Infect Dis. 2000;30:368–73. 10.1086/31365010671343

[19] Horan TC, Andrus M, Dudeck MA. CDC/NHSN surveillance definition of health care-associated infection and criteria for specific types of infections in the acute care setting. Am J Infect Control. 2008;36:309–32. 10.1016/j.ajic.2008.03.00218538699

[20] Durack DT, Lukes AS, Bright DK. New criteria for diagnosis of infective endocarditis: utilization of specific echocardiographic findings. Duke Endocarditis Service. Am J Med. 1994;96:200–9. 10.1016/0002-9343(94)90143-08154507

[21] Charlson ME, Pompei P, Ales KL, MacKenzie CR. A new method of classifying prognostic comorbidity in longitudinal studies: development and validation. J Chronic Dis. 1987;40:373–83. 10.1016/0021-9681(87)90171-83558716

[22] Lina G, Piemont Y, Godail-Gamot F, Bes M, Peter MO, Gauduchon V, Involvement of Panton-Valentine leukocidin–producing *Staphylococcus aureus* in primary skin infections and pneumonia. Clin Infect Dis. 1999;29:1128–32. 10.1086/31346110524952

[23] Diep BA, Gill SR, Chang RF, Phan TH, Chen JH, Davidson MG, Complete genome sequence of USA300, an epidemic clone of community-acquired meticillin-resistant *Staphylococcus aureus.* Lancet. 2006;367:731–9. 10.1016/S0140-6736(06)68231-716517273

[24] Harmsen D, Claus H, Witte W, Rothganger J, Claus H, Turnwald D, Typing of methicillin-resistant *Staphylococcus aureus* in a university hospital setting by using novel software for *spa* repeat determination and database management. J Clin Microbiol. 2003;41:5442–8. 10.1128/JCM.41.12.5442-5448.200314662923PMC309029

[25] Tenover FC, Arbeit RD, Goering RV, Mickelsen PA, Murray BE, Persing DH, Interpreting chromosomal DNA restriction patterns produced by pulsed-field gel electrophoresis: criteria for bacterial strain typing. J Clin Microbiol. 1995;33:2233–9.749400710.1128/jcm.33.9.2233-2239.1995PMC228385

[26] Gonzalez BE, Rueda AM, Shelburne SA III, Musher DM, Hamill RJ, Hulten KG. Community-associated strains of methicillin-resistant *Staphylococccus aureus* as the cause of healthcare-associated infection. Infect Control Hosp Epidemiol. 2006;27:1051–6. 10.1086/50792317006811

[27] Saravolatz LD, Pohlod DJ, Arking LM. Community-acquired methicillin-resistant *Staphylococcus aureus* infections: a new source for nosocomial outbreaks. Ann Intern Med. 1982;97:325–9.711462910.7326/0003-4819-97-3-325

[28] Gilbert M, MacDonald J, Gregson D, Siushansian J, Zhang K, Elsayed S, Outbreak in Alberta of community-acquired (USA300) methicillin-resistant *Staphylococcus aureus* in people with a history of drug use, homelessness or incarceration. CMAJ. 2006;175:149–54. 10.1503/cmaj.05156516804118PMC1490001

[29] Lowy FD, Aiello AE, Bhat M, Johnson-Lawrence VD, Lee MH, Burrell E, *Staphylococcus aureus* colonization and infection in New York State prisons. J Infect Dis. 2007;196:911–8. 10.1086/52093317703423

# Research article: Illicit Drug Use and Risk for USA300 Methicillin-Resistant *Staphylococcus aureus* Infections with Bacteremia

**DOI:** 10.3201/eid1609.091802

**Authors:** Kristen M. Kreisel, J. Kristie Johnson, O. Colin Stine, Michelle D. Shardell, Eli N. Perencevich, Alan J. Lesse, Fred M. Gordin, Michael W. Climo, Mary-Claire Roghmann

**Affiliations:** Author affiliations: University of Maryland, Baltimore, Maryland, USA (K.M. Kreisel, J.K. Johnson, O.C. Stine, M.D. Shardell, E.N. Perencevich, M.-C. Roghmann);; VA Maryland Health Care System, Baltimore (K.M. Kreisel, E.N. Perencevich, M.-C. Roghmann);; VA Western New York Healthcare System, Buffalo, New York, USA (A.J. Lesse);; University at Buffalo, Buffalo (A.J. Lesse);; Washington DC VA Medical Center, Washington, DC, USA (F.M. Gordin);; George Washington University, Washington (F.M. Gordin);; Hunter Holmes McGuire VA Medical Center, Richmond, VA, USA (M.W. Climo);; Medical College of Virginia at Virginia Commonwealth University, Richmond (M.W. Climo)

## Earning CME Credit

To obtain credit, you should first read the journal article. After reading the article, you should be able to answer the following, related, multiple-choice questions. To complete the questions and earn continuing medical education (CME) credit, please go to http://cme.medscape.com/viewpublication/30063. Credit cannot be obtained for tests completed on paper, although you may use the worksheet below to keep a record of your answers. You must be a registered user on Medscape.com. If you are not registered on Medscape.com, please click on the New Users: Free Registration link on the left hand side of the website to register. Only one answer is correct for each question. Once you successfully answer all post-test questions you will be able to view and/or print your certificate. For questions regarding the content of this activity, contact the accredited provider, CME@medscape.net. For technical assistance, contact CME@webmd.net. American Medical Association’s Physician’s Recognition Award (AMA PRA) credits are accepted in the US as evidence of participation in CME activities. For further information on this award, please refer to http://www.ama-assn.org/ama/pub/category/2922.html. The AMA has determined that physicians not licensed in the US who participate in this CME activity are eligible for *AMA PRA Category 1 Credits*™. Through agreements that the AMA has made with agencies in some countries, AMA PRA credit is acceptable as evidence of participation in CME activities. If you are not licensed in the US and want to obtain an AMA PRA CME credit, please complete the questions online, print the certificate and present it to your national medical association.

## Article Title: Illicit Drug Use and Risk for USA300 Methicillin-Resistant *Staphylococcus aureus* Infections with Bacteremia

## CME Questions

**1. Who is most likely to acquire soft-tissue community-acquired methicillin-resistant *Staphylococcus aureus* (MRSA) USA300 strain in an outbreak in the United States?**A. A 21-year-old male wrestlerB. A 40-year-old female prisonerC. A 29-year-old female prostituteD. A 32-year-old female illicit drug user**2. A 54-year-old, female veteran is admitted to a Veterans Affairs hospital with a suspected diagnosis of sepsis. Blood cultures are positive for MRSA USA300 strain. On the basis of this study, what are the implications of this information for directing this patient's care?**A. Illicit drug users are more likely to have an infection with an *S aureus* strain other than USA300, making a history of drug use unlikely in this patientB. Although illicit drug use is a risk factor for acquisition of infection, the percentage of infection occurring in persons without a history of drug use has increased, and this individual should not be assumed to have a history of drug useC. The percentage of infection occurring in illicit drug users increased over 4 years, and it is very likely that this individual acquired her infection through this mechanismD. Although the risk for community-acquired MRSA increased in drug-using men over the period from 2004 to 2008, no similar increase occurred in women and a history of illicit drug use is less likely in female patients

## Activity Evaluation

**Table:** 

1. The activity supported the learning objectives.				
Strongly Disagree				Strongly Agree
1	2	3	4	5
2. The material was organized clearly for learning to occur.				
Strongly Disagree				Strongly Agree
1	2	3	4	5
3. The content learned from this activity will impact my practice.				
Strongly Disagree				Strongly Agree
1	2	3	4	5
4. The activity was presented objectively and free of commercial bias.				
Strongly Disagree				Strongly Agree
1	2	3	4	5
